# Scanning MscL Channels with Targeted Post-Translational Modifications for Functional Alterations

**DOI:** 10.1371/journal.pone.0137994

**Published:** 2015-09-14

**Authors:** Irene Iscla, Robin Wray, Christina Eaton, Paul Blount

**Affiliations:** Department of Physiology, University of Texas Southwestern Medical Center at Dallas, Dallas, Texas, United States of America; Xuzhou Medical College, CHINA

## Abstract

Mechanosensitive channels are present in all living organisms and are thought to underlie the senses of touch and hearing as well as various important physiological functions like osmoregulation and vasoregulation. The mechanosensitive channel of large conductance (MscL) from *Escherichia coli* was the first protein shown to encode mechanosensitive channel activity and serves as a paradigm for how a channel senses and responds to mechanical stimuli. MscL plays a role in osmoprotection in *E*. *coli*, acting as an emergency release valve that is activated by membrane tension due to cell swelling after an osmotic down-shock. Using an osmotically fragile strain in an osmotic down-shock assay, channel functionality can be directly determined *in vivo*. In addition, using thiol reagents and expressed MscL proteins with a single cysteine substitution, we have shown that targeted post-translational modifications can be performed, and that any alterations that lead to dysfunctional proteins can be identified by this *in vivo* assay. Here, we present the results of such a scan performed on 113 MscL cysteine mutants using five different sulfhydryl-reacting probes to confer different charges or hydrophobicity to each site. We assessed which of these targeted modifications affected channel function and the top candidates were further studied using patch clamp to directly determine how channel activity was affected. This comprehensive screen has identified many residues that are critical for channel function as well as highlighted MscL domains and residues that undergo the most drastic environmental changes upon gating.

## Introduction

Mechano-transduction, the ability to sense and respond to mechanical stimuli, is thought to underlie the senses of touch, hearing, balance, proprioception as well as fundamental physiological processes, such as osmoregulation, vascular regulation, bladder control, and ischemia. Mechanosensitive channels are proposed to be the molecular entities underlying this sense at the cellular level, and are present in essentially all living organism from archaebacteria to plants to mammals. The first cloned mechanosensitive channel is the bacterial mechanosensitive channel of large conductance, MscL, and it is to date one of the best studied, thus serving as a paradigm of how mechanosensors sense and respond to membrane stretch [1]. Although there are no obvious homologies between bacterial mechanosensitive (MS) channels and candidates for eukaryotic MS channels, there are structural similarities, including an amphipathic helix adjacent to the pore running parallel to the cytoplasmic side of the membrane, and a series of charges at the cytoplasmic region of a non-pore-forming transmembrane domain. In addition, channels from both kingdoms have shown modulation by some amphipathic compounds such as lysophosphatidylcholine (LPC) and arachidonic acid (AA), which alter the membrane lipid tension profile.

Mechanosensitive channels in bacteria serve a role in osmoprotection. MscL and the mechanosensitive channel of small conductance MscS have a concerted role in helping bacteria cope with sudden decreases in the osmolarity of their environment or osmotic down-shock. These channel’s large conductance of approximately 3 and 1 nS respectively allow for the rapid flow of solutes to reach a new osmotic balance and save the cells from lysis. They have a redundant role with only the double null strain being osmotically fragile. This phenotype can be rescued with the expression, in trans, of either functional MscL or MscS and gives a sensitive screen to detect changes in channel function *in vivo*.

**Fig 1:**
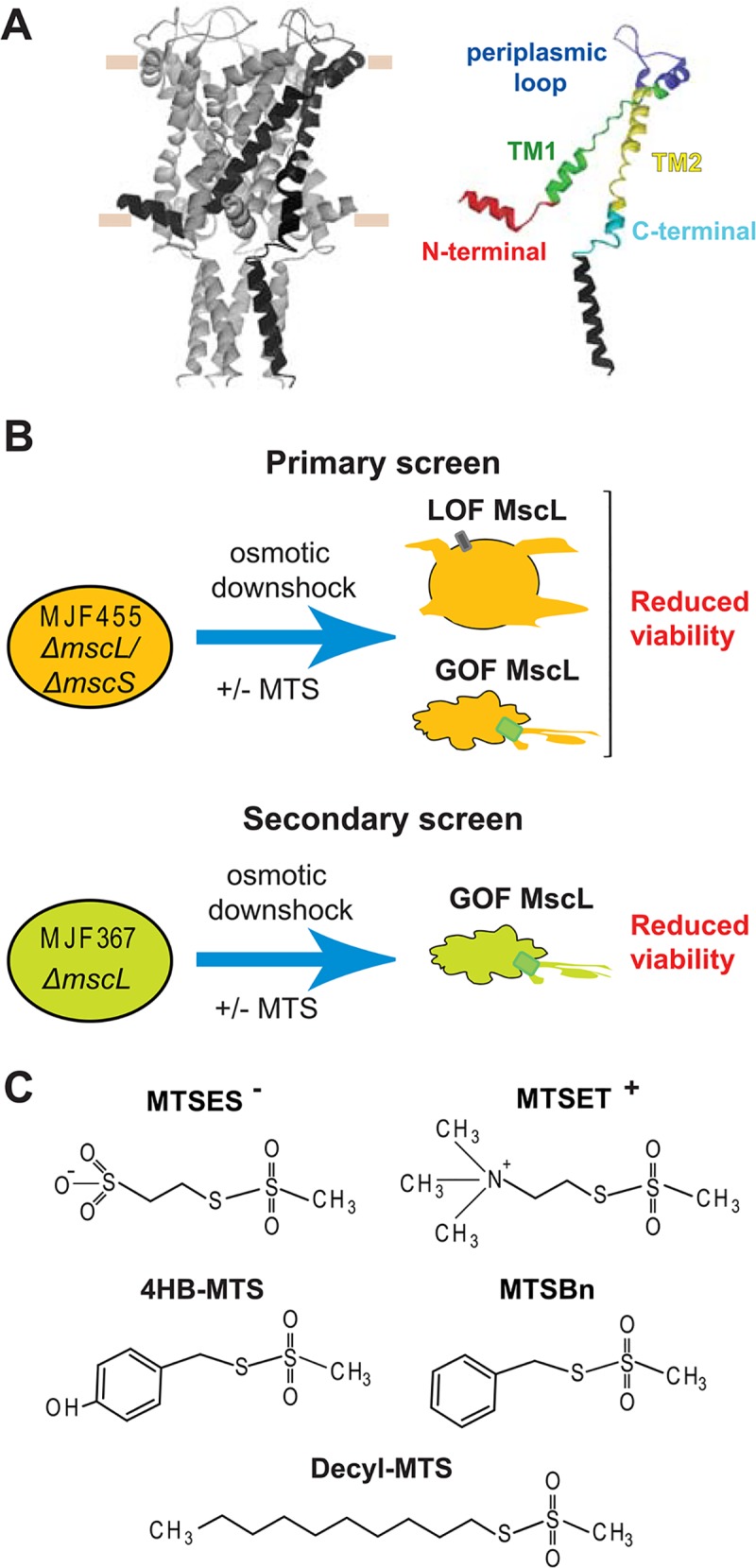
*In vivo* screens to determine MscL activity changes after post-translational modifications. (A) The structure of *E*. *coli* MscL in its closed state [16], generously provided by Ben Corry, is shown in a side view, with a subunit highlighted for clarity. The approximate location of the lipid membrane headgroups is marked by horizontal tan lines. The domains, in which the study has been divided, are highlighted in an isolated subunit using different colors. (B) A schematic description of the *in vivo* screens used to study the activity of the MscL cysteine substituted channels before and after post-translational modification with different MTS reagents. In a primary screen, the osmotically fragile strain MJF 455 is osmotically shocked with or without the MTS reagents present during the osmotic down-shock. Channels with reduced sensitivity (Loss of function LOF) or increased sensitive to tension (gain of function GOF), lead to cells with reduce viability in the primary *in vivo* screens. A secondary screen is done to distinguish between these two phenotypes. The secondary screen consists in MJF367 strain (not osmotically fragile) osmotically shocked with the MTS reagents present in during the osmotic down-shock. In the secondary screens a reduced viability indicates more sensitive channel or GOF. (C) The structure of the sulfhydryl reagents 2-sulfonatoethyl methanethiosulfonate sodium salt (MTSES^-^), ethyl methanethiosulfonate Bromide (MTSET^+^), 4-hydroxybenzyl methanethiosulfonate (4-HB-MTS), benzyl methanethiosulfonate (MTSBn), and decyl methanethiosulfonate (decyl-MTS), 2-(Trimethylammonium) are shown.

The crystal structure of a closed state of *M*. *tuberculosis* MscL was solved by the Rees group revealing a homopentameric protein, with both N- and C-terminal predicted to be on the cytoplasmic side [2, 3]. The N-terminal domain appears as a helix running parallel to the cytoplasmic membrane, the first transmembrane domain (TM1) forming the pore, the second transmembrane domain (TM2) in close contact with the lipids, the C-terminal domain forming a cytoplasmic helical bundle (Fig 1A). This bundle remains in place during channel gating [4]. Unfortunately, an open-state crystal structure of the channel has proved elusive to date, but a structural model of an open MscL based on electro paramagnetic resonance (EPR) studies has been generated, predicting a tilting of the two transmembrane domains during channel gating [5].

The large conductance of 3nS and molecular sieving experiments predicting a 30 Å pore [6] suggest that the MscL channel undergoes a large conformational change upon channel gating with many residues and domains changing their local environment. Presumably, encouraging or discouraging these movements by making specific individual residues more hydrophilic or hydrophobic would lead to changes in the functional properties of the channel.

One of the great advantages of studying microbial channels is the ability to use screens to directly assess the functional properties of expressed proteins. Here we utilize such a screen to determine the activity of MscL *in vivo* by assessing phenotypic changes, and thus determine which post-translational changes in MscL modified its function. Using a library of MscL cysteine mutants spanning 113 of the 136 residues, we studied the effects of five different post-translational modifications *in vivo*, thus totaling 678 potential changes, including the original cysteine substitutions. Modifications that yielded interesting phenotypic changes have been further studied by patch clamp to determine their effects on channel activities. The data not only identify critical residues, but also make predictions of how the channel changes conformation upon channel gating.

## Materials and Methods

### Strains and cell growth

All mutants were generated using the Mega Primer method as described [7] and were inserted in the pB10b or pB10d expression constructs [8–10]. *E*. *coli* strain PB104 (*ΔmscL*::Cm) [9] was used for electrophysiological and for stationary growth experiments. The *E*. *coli* FRAG-1 [11] derivative strain, MJF455 *ΔmscL*::Cm, *ΔmscS* and MJF367 *ΔmscL*::*Cm* [12] were used experiments after osmotic down-shock and electrophysiology. Cultures were routinely grown in Lennox Broth media (LB) (Fisher Scientific, Pittsburgh, PA), or citrate-phosphate defined medium (CphM) consisting of (per liter: 8.57 g of Na2HPO4, 0.87 g of K2HPO4, 1.34 g of citric acid, 1.0 g of NH4SO4, 0.001 g of thiamine, 0.1 g of Mg2SO4.7H2O, 0.002 g of (NH4)2SO4.FeSO4.6H2O) plus 100 μg/mL ampicillin, in a shaker-incubator at 37°C and rotated at 250 cycles per minute. Protein expression was induced by addition of 1 mM isopropyl-β-D-thiogalactopyranoside (IPTG) (Anatrace Inc, Maumee, OH).

Sulfhydrylreagents 4-hydroxybenzyl methanethiosulfonate (4-HB-MTS), benzyl methanethiosulfonate (MTSBn), decyl methanethiosulfonate (decyl-MTS), 2-(Trimethylammonium) ethyl methanethiosulfonate Bromide (MTSET^+^) and 2-sulfonatoethyl methanethiosulfonate sodium salt (MTSES^-^) were obtained from Toronto Research Chemicals (North York, ON, Canada). MTS reagents were dissolved in methanol or ethanol, according to manufacturer’s recommendation, to a 100mM concentration and stored at -20°C in argon; they were diluted to their final concentration right before using.

### *In Vivo* Functional Assays

Viability experiments were done using the *E*. *coli* MJF455 and MJF367 strains. Colonies were grown overnight at 37°C in citrate phosphate medium plus 1 mM ampicillin. The overnight culture was diluted 1:20 in this defined medium, grown for 1h and then diluted to an OD_600_ of 0.05 in the same medium supplemented with 0.5M NaCl. Cultures were induced at an OD_600_ 0.2, for 30 minutes with 1 mM IPTG. The induced cultures were diluted 1:20 into (i) citrate-phosphate medium containing 0.5 M NaCl (mock shock); (ii) water (osmotic down-shock). Cells were then incubated at 37°C for 20 min, and then six consecutive 1:10 serial dilutions were made in medium containing either no salt (for the osmotic down-shock conditions) or 0.5 M NaCl (for the mock-shock conditions). These diluted cultures were plated and grown overnight and the colony-forming units were counted and averaged per experiment. To assess the effect of the different MTS reagents in cell viability they were added to the shock solution at the following concentrations: 50μM 4-HB-MTS, 5–10μM MTSBn, 10μM Decyl-MTS, 1mM MTSET^+^ and 2mM MTSES^-^.

### Electrophysiology

*E*. *coli* giant spheroplasts were generated and used in patch-clamp experiments as described previously [13]. Excised, inside-out patches were examined at room temperature under symmetrical conditions using a buffer comprised of 200 mM KCl, 90 mM MgCl_2_, 10 mM CaCl_2_, and 5 mM HEPES pH 6 (Sigma, St. Louis, MO). MTS reagents were added to the bath chamber or backfilled in the patch pipette at the following final concentrations: 50μM 4-HB-MTS, 100μM MTSBn, 10μM Decyl-MTS, 1mM MTSET^+^ and 2mM MTSES^-^.

Recordings were performed at –20 mV (positive pipette). Data were acquired at a sampling rate of 20 kHz with a 5 kHz filter using an AxoPatch 200B amplifier in conjunction with Axoscope software (Axon Instruments, Union City, CA). A piezoelectric pressure transducer (World Precision Instruments, Sarasota, FL) was used to monitor the pressure throughout the experiments. MscL pressure threshold was defined as the pressure at which openings were observed at least every 0.5 seconds. Measurements before and after treatment with MTS reagents were compared in the same patch as previously described [14]. Analysis was performed using Clampfit10 from Pclamp10 (Axon Instruments, Union City, CA).

## Results

### *In vivo* screens

As the MscL channel gates, it changes conformation and several individual residues undoubtedly change their local environment with some going into more hydrophilic environments, other going into more hydrophobic. If these changes are encouraged or inhibited, the function of the channel should be altered. Under the hypothesis that such changes in MscL channel function can be detected using *in vivo* assays, we used a cysteine library (residues S2-L114, excluding only the C-terminal helical bundle, which has little if any functional role [4, 10, 15], and post-translationally modified the channel with five different substitutions by using sulfhydryl probes. Each probe can potentially react with the cysteine, and since they have different charges or different affinities to the membrane, they conferred these properties to the cysteine substituted site. Therefore, by determining how these changes affect channel activity *in vivo* and *in vitro*, we can infer what environmental changes take place during channel gating at that site (Fig 1).

A combination of two *in vivo* assays was used to determine the phenotype of the modified channels. For the primary *in vivo* screen, we used the *E*. *coli* MJF 455 strain, null for two major mechanosensitive channels. This is an osmotically fragile strain, whose viability is severely compromised upon a sudden reduction of the osmolarity of the media (osmotic down-shock) (S1A Fig). This phenotype can be rescued by expression *in trans* of WT MscL and to a different extent by mutated MscL channels depending on the severity of the mutation on channel activity. Although this is a very sensitive assay, two opposite channel phenotypes can lead to an observed reduced viability: channels that require very high pressures to gate, higher than the lytic pressure of the membrane, thus phenotypically loss of function (LOF), or channels that gate at lower than normal pressures and leak the contents of the cells, or gain of function (GOF) (Fig 1B). To distinguish between these two possible phenotypes, a second *in vivo* screen can be used by evaluating the viability of a non-osmotically fragile strain MJF 367 (MscL null but still containing MscS), with or without the MTS reagents present during the osmotic down-shock. Only the misgating of MscL at lower than normal tensions, i.e. a GOF phenotype, causes a reduced viability; LOF phenotypes will not cause a reduced viability in these secondary screens because the cell contains MscS and thus is not osmotically fragile.

We used five MTS reagents in the primary screen. Three of them are largely hydrophobic, but with different degrees of hydrophobicity; MTSBn, which simply contains a phenyl group, 4HB-MTS, which has a tyrosine-like group and a slightly higher preference for the lipid-aqueous interface [17], and decyl-MTS, which has a ten carbon long lipid-like fatty acid chain that could drag the modified site to the membrane [18]. The other two are the charged MTS probes MTSET^+^ and MTSES^-^, with positive and negative charges respectively, (Fig 1C).

The ability of each mutated channel to rescue the osmotically fragile *E*. *coli* strain MJF 455 was assayed in an *in vivo* assay. Briefly, bacterial cells expressing each channel were grown in a high osmolarity media and subsequently diluted in media of the same osmolarity (non-shocked) or into water to produce an osmotic down-shock; because the cysteine mutation itself could effect a LOF or GOF phenotype, some of the mutants demonstrated a loss-of-viability phenotype upon this osmotic down-shock (S1A–S1E Fig). In the same set of experiments, samples were also shocked in the presence of the five different MTS reagents described above. For every mutant the plots represent the change in viability between shocked and shocked in the presence of the MTS reagents. Negative values indicate that the expressed channel malfunctions after modification with a probe, while a positive value means that the MTS treatment improves the functionality of that cysteine mutant, and thus its ability to better rescue the osmotically fragile strain. To distinguish modifications that yielded the greatest changes in viability, we used a benchmark of a 50% change in viability. For the clarity of the paper we present the data divided into protein domains.

### The N-terminal region of *E*. *coli* MscL: S1 and S1-TM1 linker

As described above, the effect of the cysteine substitutions in MscL channel activity *in vivo* was initially evaluated with no MTS reagents present during the osmotic down-shock. Of the 17 residues included in the N-terminal region (residues S2 to D18), 9 showed a lower viability due to a decrease in sensitivity or LOF phenotype and 3 appear to be due to a GOF phenotype (S1A Fig) [7, 8]. Interestingly, this is the region in which the cysteine substitutions had the greatest effect on channel activity, thus highlighting its important role in channel gating [8].

**Fig 2:**
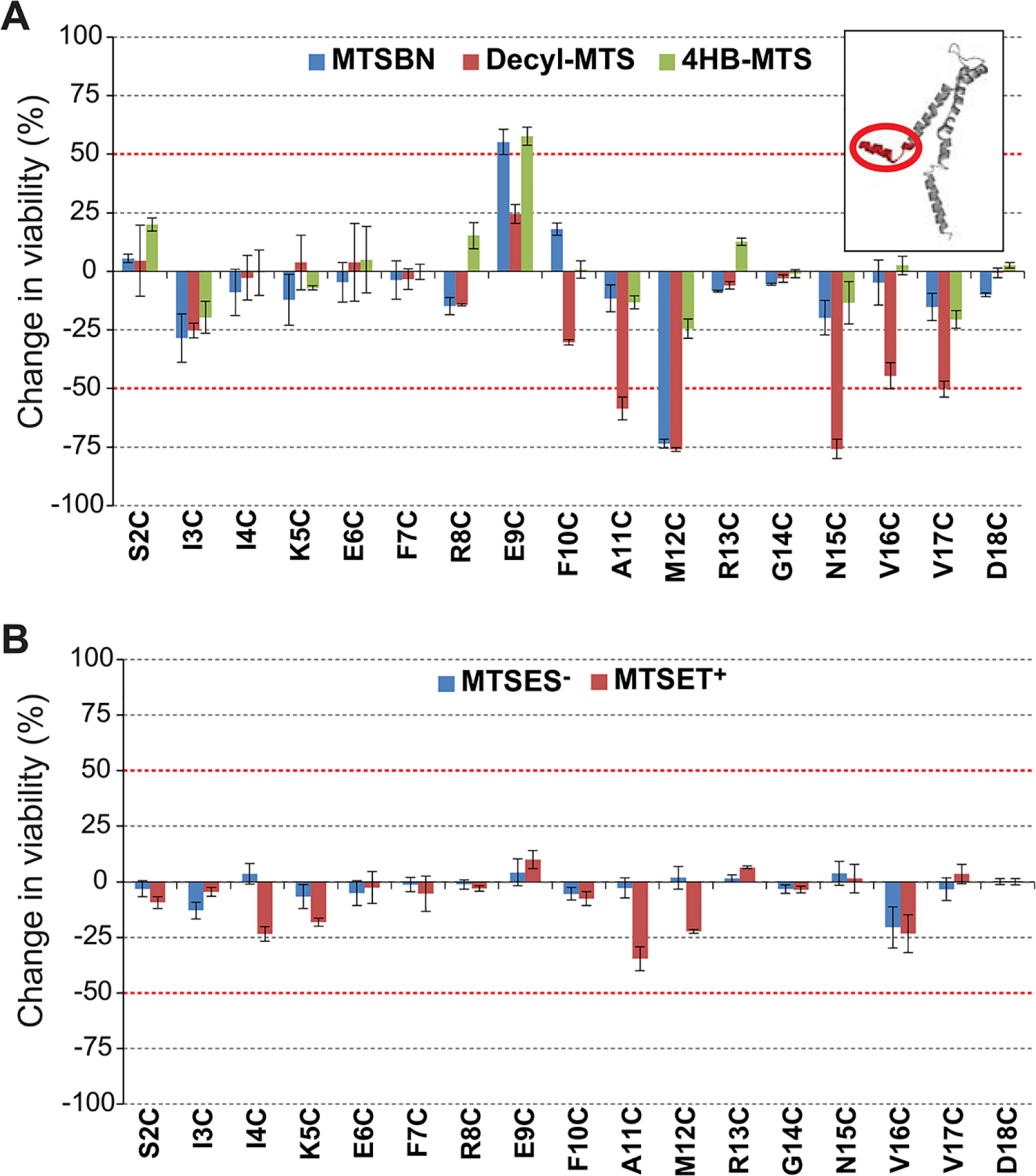
Effects of post translational modifications on the N-terminal domain of MscL determined by *in vivo* screens. The viability of the osmotically fragile strain MJF455 expressing MscL cysteine substituted mutants from residues S2 to D18 (insert), was measured after an osmotic down-shock. The graphs show the differences in viability between non-treated and post-transnationally modified channels with (A) the hydrophobic MTS reagents MTSBn (blue), decyl-MTS (red) or 4HB-MTS (green) or (B) the negatively charged MTSES^-^ (blue) and positively charged MTSET^+^ (red). The red grid line indicates a ± 50% change that was used as a threshold for further studies.

**Fig 3:**
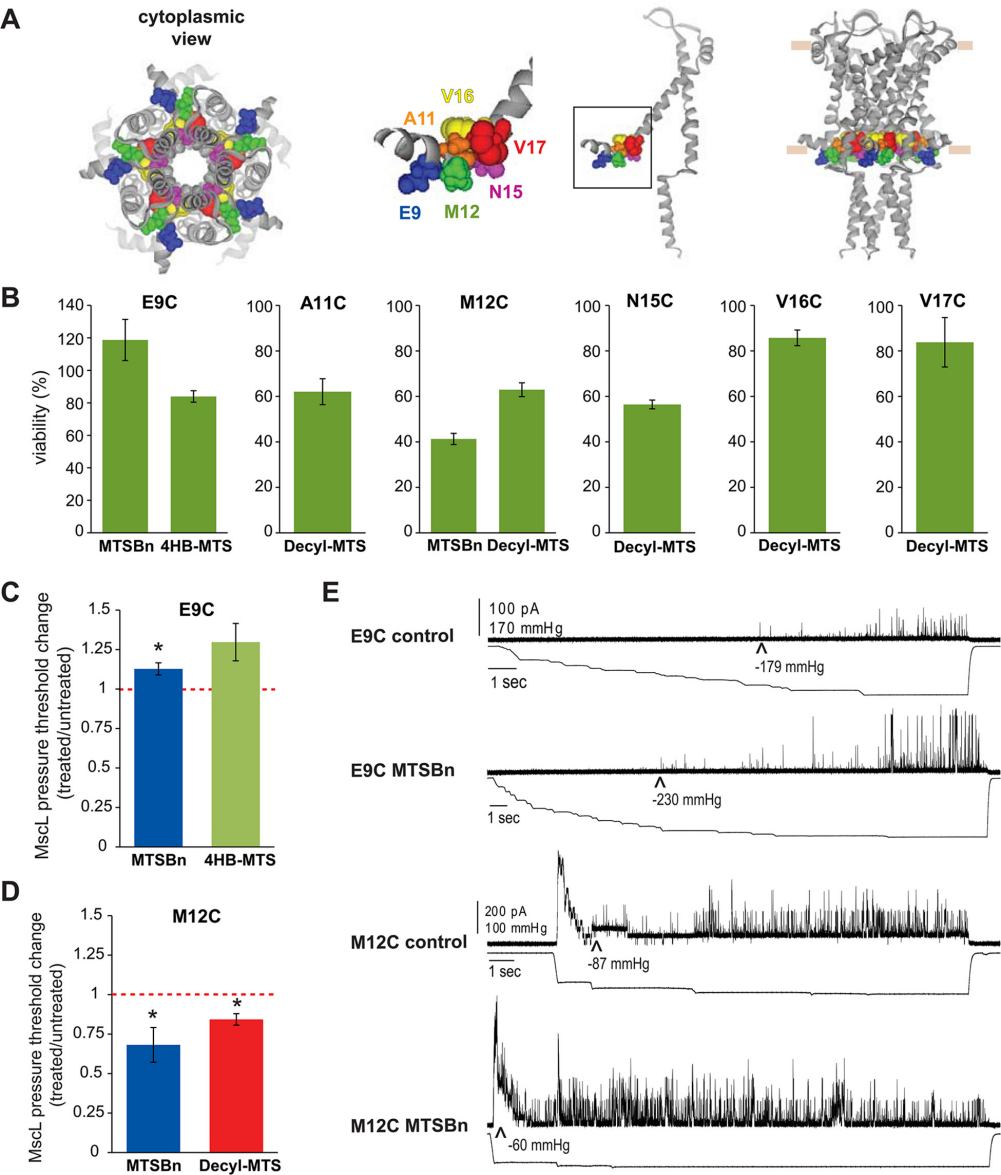
Functional changes by substitutions in the N-terminal domain of MscL, determined by *in vivo* and patch clamp experiments. (A) The location of the residues showing changes in viability upon post-translational modifications is highlighted in the closed structure of *E*. *coli* MscL. From right to left: a pentameric MscL is shown in a side view followed by a single subunit and a close up of the region and a cytoplasmic view of the pentameric channel. (B) Viability of MJF 367 (*mscL* ^-^) shocked in the presence of the indicated MTS reagent is shown for each individual MscL mutant. (C) The changes in the pressure threshold required to gate E9C MscL caused by treatment with MTS reagents is graphed as the ratio between before and after modification of the same patch. The red line indicated no change. (D) The changes in the pressure threshold required to gate M12C MscL caused by MTS reagents is graphed as the ratio between before and after modification of the same patch. The red line indicates no change. (E) Representative traces of E9C MscL and M12C before (control) and after treatment with MTSBn. The upper traces correspond to the current and the lower traces the negative pressure applied to the patch.

Post-translational modification with the hydrophobic MTS reagents MTSBn, decyl-MTS, and 4HB-MTS, produced greater than 50% changes in viability after an osmotic down-shock for channels modified at residues E9, A11, M12, N15, V16 and V17 (Figs 2A and 3A). Interestingly, E9 showed an increase in viability as opposed to a decrease. These data would be consistent with the GOF phenotype observed for the untreated E9C mutated protein, as assayed by osmotic down-shock (see S1 Fig and [8]) being rectified by the modifications. No changes in the viability were observed when shocked in the presence of MTSES^-^ and MTSET^+^ (Fig 2B).

Secondary screens were performed in order to determine which channel phenotype caused the observed changes in viability. As explained in Fig 1B, a reduced viability in the secondary screens are the result of more sensitive channels, a GOF phenotype, while an unchanged viability indicates a LOF phenotype. Using the strain MJF367, which is only null for MscL and thus not osmotically fragile, hydrophobic substitutions at residues A11, M12 and N15 led to reduced viability, demonstrating that these modifications lead to a GOF phenotype (Fig 3A and 3B). In contrast with these results, modification of V16 and V17 residues gave no indication of leading to a GOF phenotype after modification in the secondary screens, suggesting they led to a LOF phenotype. As discussed above, E9C is a GOF independent of any modification and it appears this phenotype is remediated by MTSBn and 4HB-MTS; these data could indicate that these modifications lead to a more wild type functional channel, or a LOF channel.

We chose two of the more interesting mutated channels for patch clamp analysis at the single channel level in native membranes derived from giant spheroplasts. As we had predicted above, an increase in the pressure was needed to gate the E9C channel after incubation with MTSBn and decyl-MTS (Fig 3C), consistent with remediating the GOF channel phenotype. In contrast, less pressure was needed to gate M12C after treatment with MTSBn or decyl-MTS (Fig 3D). Representative traces for both mutants are shown in Fig 3F, before (control) and after adding MTSBn to the bath, with the threshold pressure indicated under the arrowheads. These data are consistent with and largely confirm the *in vivo* results and interpretations.

### The first transmembrane domain: TM1

The effect of cysteine substitutions on MscL channel activity *in vivo* was studied for the pore forming domain TM1 (residues L19-G46). Of the 25 residues included in this region 3 showed a lower viability due to a decrease in sensitivity or LOF phenotype and 6 due to a GOF phenotype (S1B Fig)[7]; the latter are presumably because of the disruption of the hydrophobic lock that stabilizes the closed state of the channel [10].

**Fig 4:**
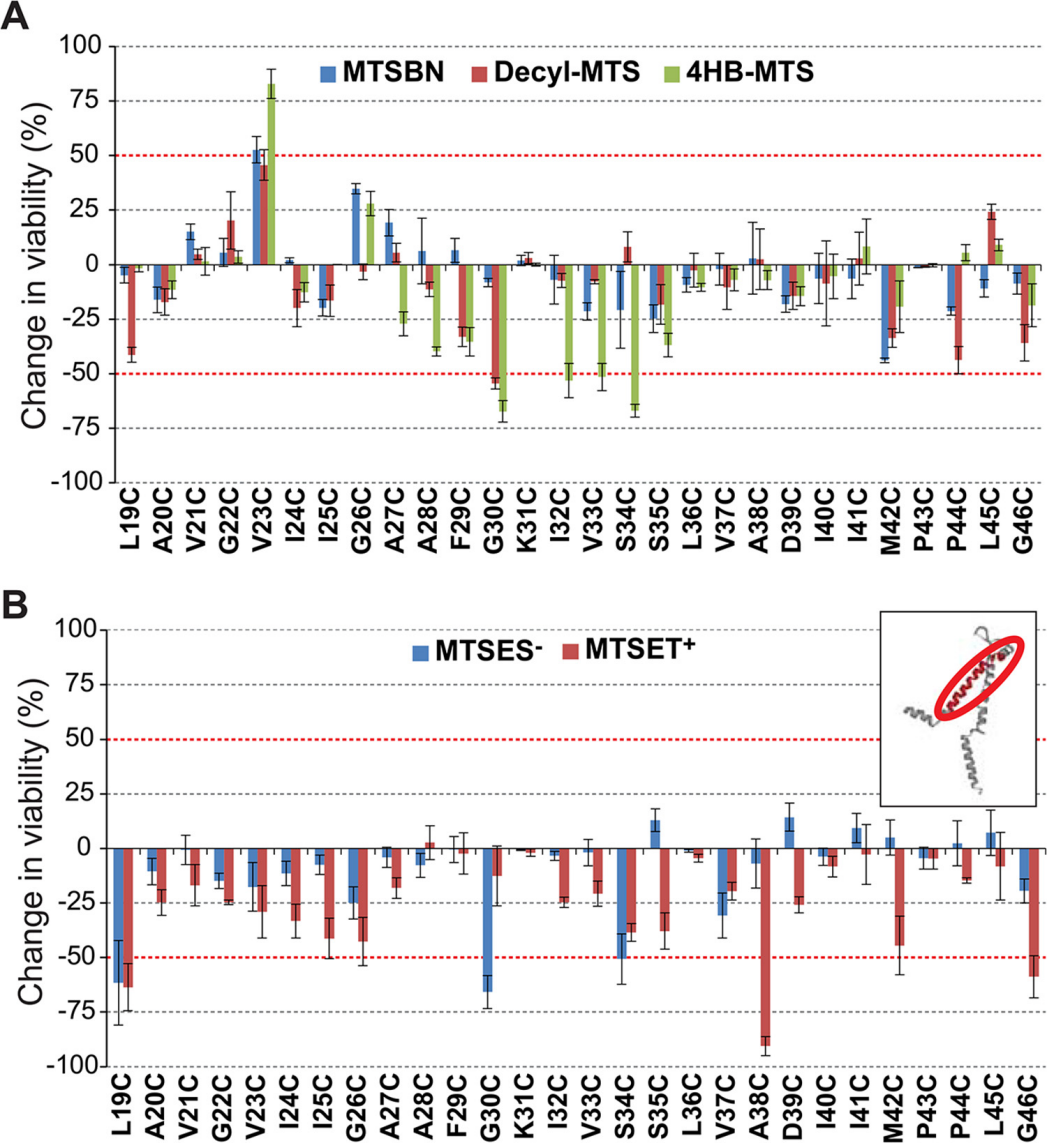
Effects of post translational modifications on the TM1 domain of MscL determined by *in vivo* channel activity. The viability of the osmotically fragile strain MJF455 expressing MscL cysteine substituted mutants from residues L19 to G46 (insert), was measured after an osmotic down-shock. The graphs show the differences in viability between non-treated and post-transnationally modified channels with (A) the hydrophobic MTS reagents MTSBn (blue), decyl-MTS (red) or 4HB-MTS (green) or (B) the negatively charged MTSES^-^ (blue) and positively charged MTSET^+^ (red). The red grid line indicates a ± 50% change that was used as a threshold for further studies.

Post-translational modification in this region with the hydrophobic MTS reagents MTSBn, decyl-MTS, and 4HB-MTS, produced changes greater than 50% in their viability after an osmotic down-shock for mutants V23C, G30C, V33C, and S34C (Fig 4A). Again one of the mutated channels, V23C, showed an increased viability upon modification, suggesting some sort of remediation of the GOF phenotype induced by the cysteine mutation (see discussion of E9C above). Changes in the viability were observed for mutants L19C, G30C, A38C and G46C, when shocked in the presence of MTSES^-^ and MTSET^+^ (Fig 4B).

**Fig 5:**
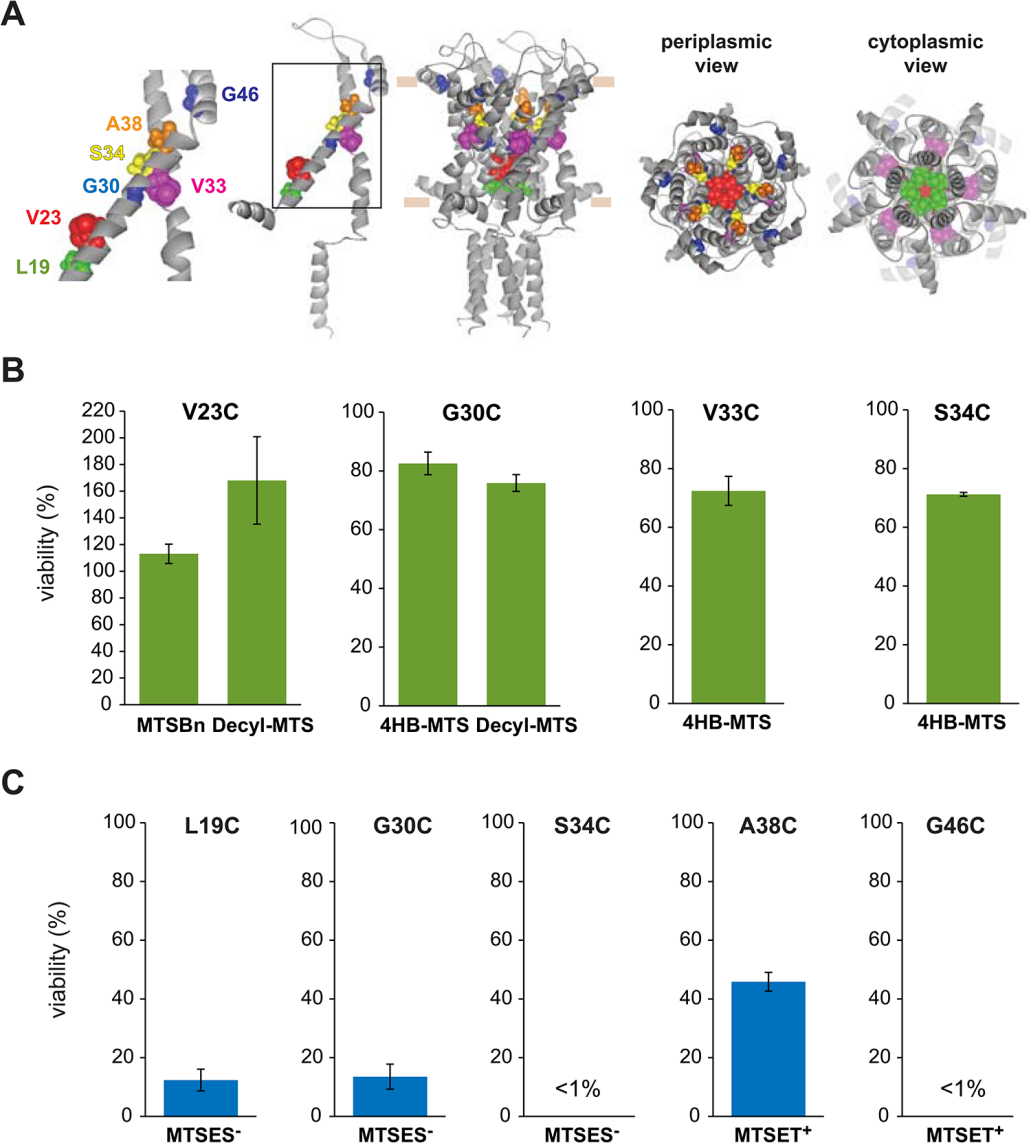
Functional changes by substitutions in the TM1 domain of MscL, determined by *in vivo* and patch clamp experiments. (A) The location of the residues showing changes in viability upon post-translational modifications is highlighted in the closed structure of *E*. *coli* MscL. From right to left: a pentameric MscL is shown in cytoplasmic view, a periplasmic view, and a side view where the approximate location of the membrane is shown. A single subunit and a close up of the region with the labeled residues is on the left. (B) Viability of MJF 367 (*mscL* ^-^) cells, shock in the presence of the indicated hydrophobic MTS reagent is shown for each individual MscL mutant. (C) Viability of MJF 367 osmotically shocked with charged MTS reagents is shown for the indicated cysteine mutants.

To determine which channel phenotype caused the observed changes in viability, secondary screens were performed. As discussed above, the interpretation of data for V23C is an apparent remediation of the GOF phenotype by the hydrophobic probes. For mutants G30C, V33C and S34C, the hydrophobic substitutions didn’t affect the viability in the assay using the MJF367 MscL-null strain; on the other hand, charged substitutions yielded dramatic decreases in viability for L19C, G30C, S34C, A38C and G46C with this assay (Fig 5A and 5B). In essence, the *in vivo* data for TM1 is consistent with a “hydrophobic lock” hypothesis which states that it is largely the transient exposure of hydrophobic residues in TM1 to an aqueous environment that is the primary energy barrier for channel opening [10, 19–22].

The TM1 domain has been previously well studied and many MscL channels with modifications in this domain, including many of the sites identified in these screens, have previously been patched with results supporting the “hydrophobic lock”. One potential complication of working with channels with cysteine mutations in this domain is that while they may give GOF phenotypes *in vivo*, where the environment is highly reduced, they may form inter-subunit disulfide bridges that lock the channel closed in patch clamp at ambient redox. This has been previously reported [7], and an example is shown for V23C treated with MTSBn and decyl-MTS in S2 Fig.

### The periplasmic loop

The effect of cysteine substitutions on MscL channel activity *in vivo* was studied for the periplamic loop (residues L47-H74). Of the 28 residues included in this region, 3 showed a lower viability due to a decrease in sensitivity or LOF phenotype and 2 due to a GOF phenotype (S1C Fig and [7]).

**Fig 6:**
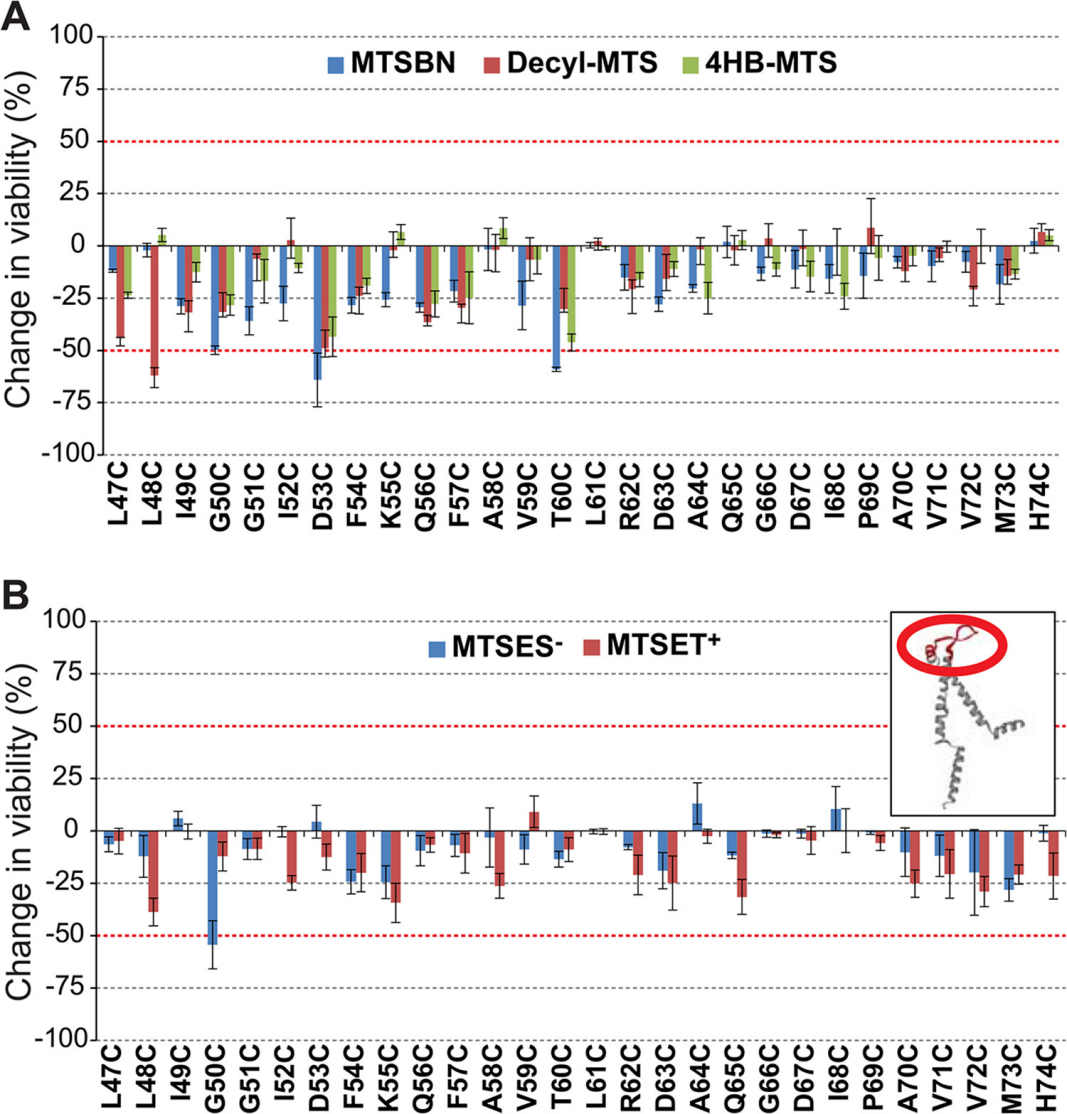
Effects of post translational modifications on the periplasmic loop of MscL determined by *in vivo* channel activity. The viability of the osmotically fragile strain MJF455 expressing MscL cysteine substituted mutants from residues L47 to H74 (insert), was measured after an osmotic down-shock. The graphs show the differences in viability between non-treated and post-transnationally modified channels with (A) the hydrophobic MTS reagents MTSBn (blue), decyl-MTS (red) or 4HB-MTS (green) or (B) the negatively charged MTSES^-^ (blue) and positively charged MTSET^+^ (red). The red grid line indicates a ± 50% change that was used as a threshold for further studies.

Post-translational modification in this region with the hydrophobic MTS reagents MTSBn, decyl-MTS, and 4HB-MTS, caused changes in viability after an osmotic shock for mutants L48C, G50C, D53C, and T60C (Fig 6A). A similar effect was observed for G50C when shocked in the presence of MTSES^-^ (Fig 6B).

**Fig 7:**
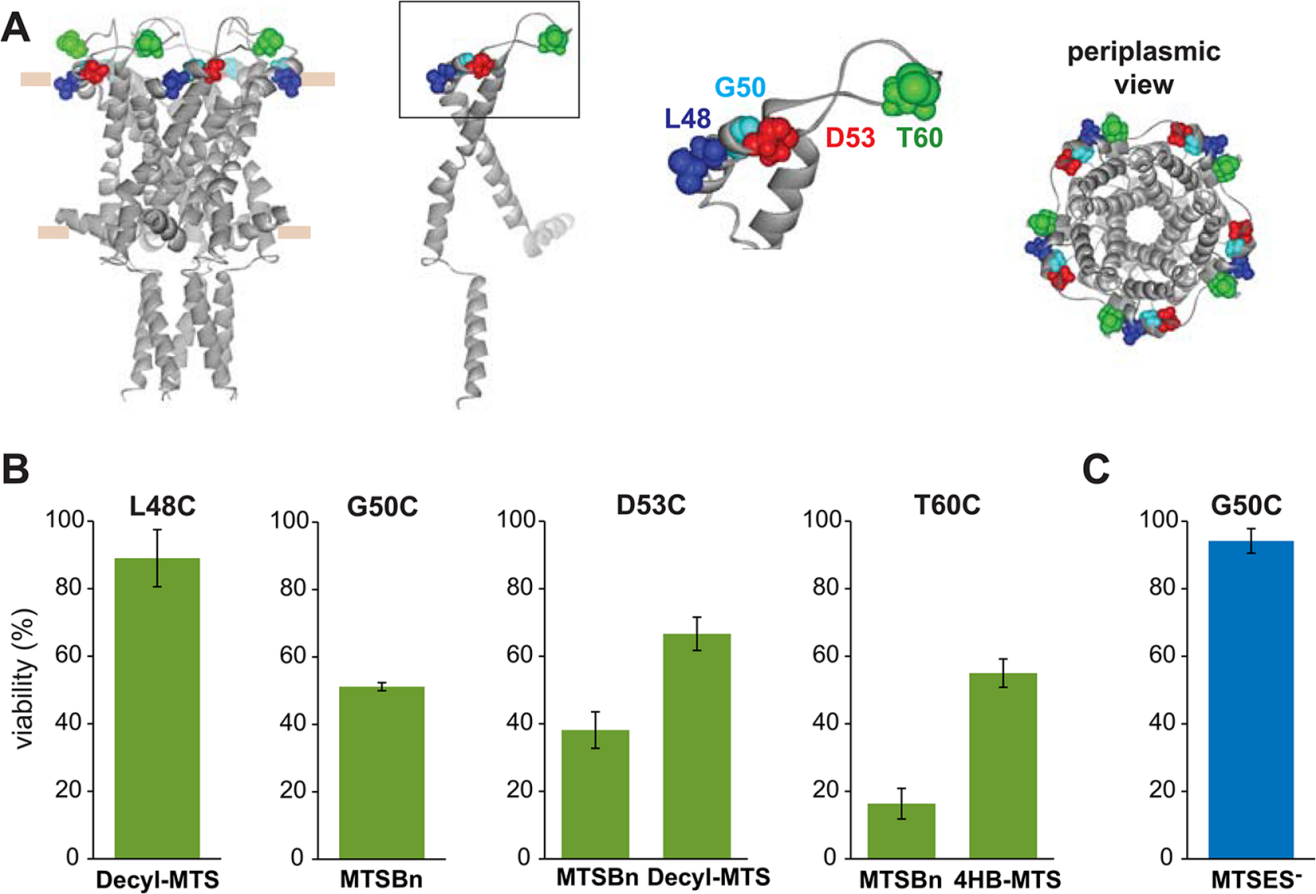
Functional changes by substitutions in the periplasmic loop of MscL, determined by *in vivo* and patch clamp experiments. (A) The location of the residues showing changes in viability upon post-translational modifications is highlighted in the closed structure of *E*. *coli* MscL. From right to left: a pentameric MscL is shown in cytoplasmic view, a periplasmic view, and a side view where the approximate location of the membrane is shown. A single subunit and a close up of the region with the labeled residues is on the left. (B) Viability of MJF 367 (*mscL* ^-^) shock in the presence of the indicated MTS reagent is shown for each individual MscL mutant, for hydrophobic or (C) charged MTS reagents.

To determine which channel phenotype caused the observed changes in viability, secondary screens were performed. In these screens, only small if any decreases in viability were observed, suggesting that none of the modifications lead to a severe GOF phenotype, with the exception of T60C treated with MTSBn (Fig 7A and 7B).

### The second transmembrane domain: TM2

The effect of cysteine substitutions on MscL’s channel activity *in vivo* was studied for the second transmembrane domain TM2, (residues Y75-N100). I92C MscL was excluded from this study because expression of this mutant caused a severe reduction on bacterial growth [7]. Of the 25 residues included in these region 7 showed a lower viability due to a decrease in sensitivity or LOF phenotype and 2 due to a GOF phenotype (S1D Fig and (Levin and Blount, 2004)).

**Fig 8:**
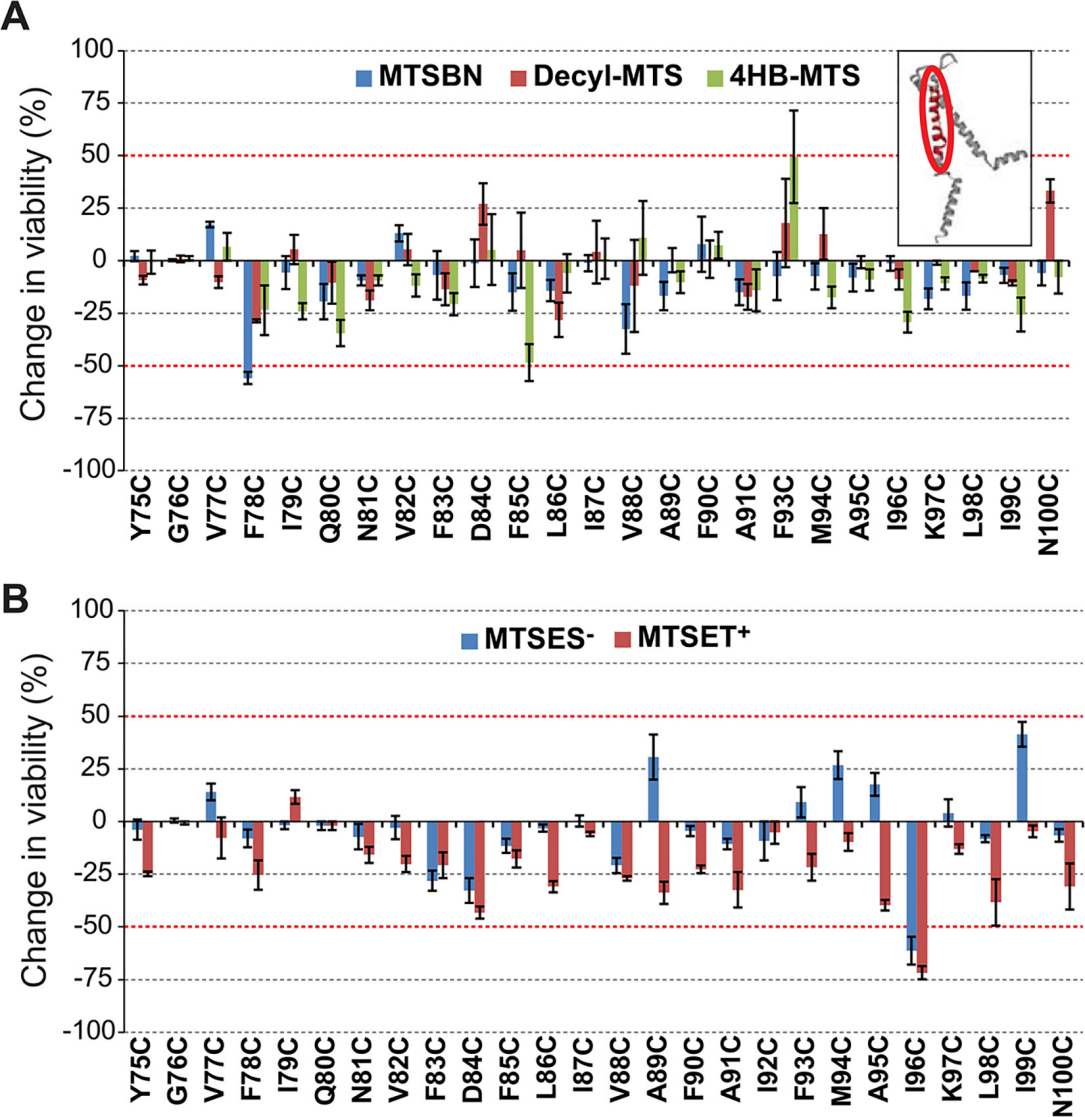
Effects of post translational modifications on the TM2 domain of MscL determined by *in vivo* channel activity. The viability of the osmotically fragile strain MJF455 expressing MscL cysteine substituted mutants from residues Y75 to N100 (insert), was measured after an osmotic down-shock. The graphs show the differences in viability between non-treated and post-transnationally modified channels with (A) the hydrophobic MTS reagents MTSBn (blue), decyl-MTS (red) or 4HB-MTS (green) or (B) the negatively charged MTSES^-^ (blue) and positively charged MTSET^+^ (red). The red grid line indicates a ± 50% change that was used as a threshold for further studies.

Post-translational modifications in this region with the hydrophobic MTS reagent MTSBn, changed the viability of F78C more than 50% when compared to only osmotic down-shock (Fig 8A). Changes in the viability of cells expressing I96C MscL were observed when shocked in the presence of MTSES^-^ and MTSET^+^ (Fig 8B).

**Fig 9:**
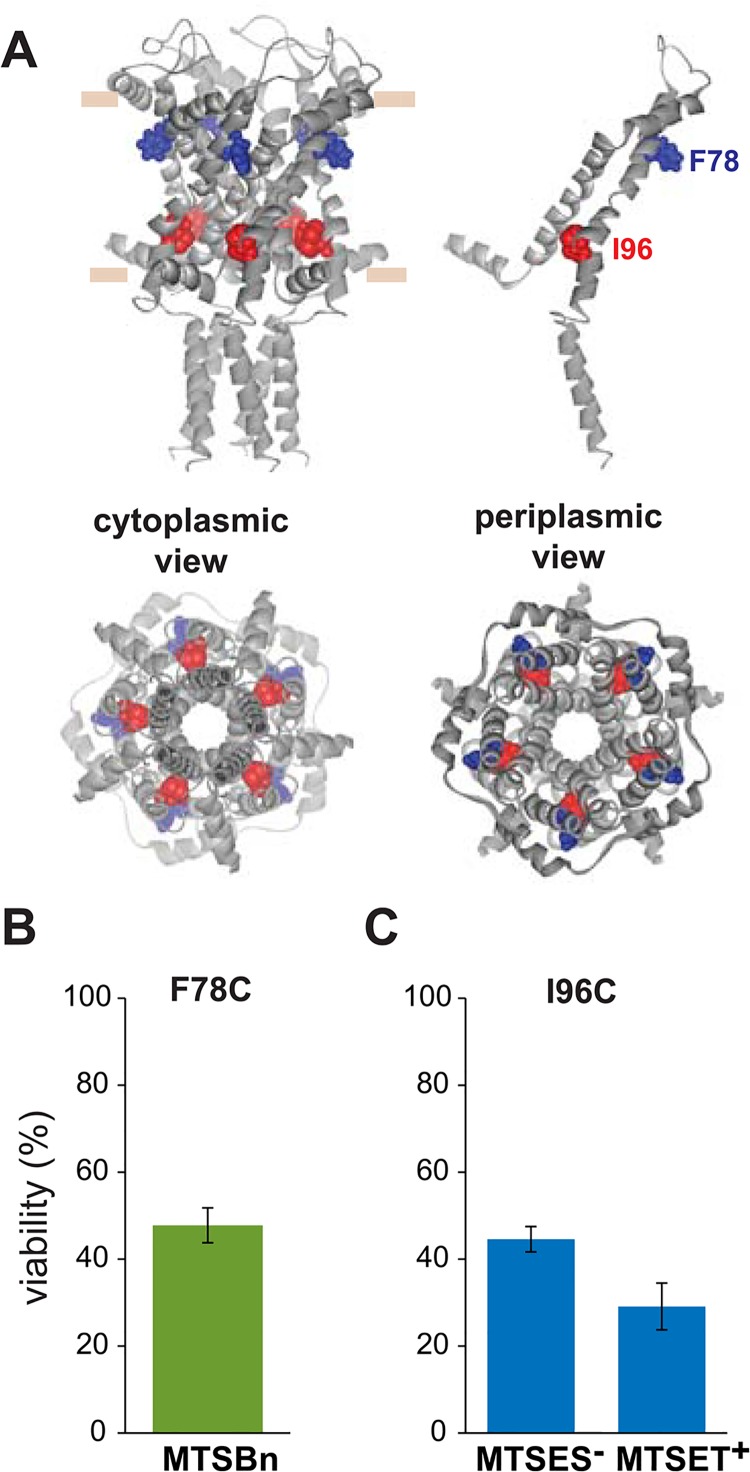
Functional changes by substitutions in the TM2 domain of MscL, determined by *in vivo* and patch clamp experiments. (A) The location of the residues showing changes in viability upon post-translational modifications is highlighted in the closed structure of *E*. *coli* MscL. A pentameric MscL is shown side view where the approximate location of the membrane marked, followed by single subunit with the labeled residues. Cytoplasmic and periplasmic views are also shown below. (B) Viability of MJF 367 (*mscL* ^-^) cells, shock in the presence of the indicated MTS reagent is shown for each individual MscL, for hydrophobic or C. charged MTS reagents.

To determine which channel phenotype caused the observed changes in the viability, secondary screens were performed. MTSBn substitution at residue F78 and charged substitutions at residue I96, effected GOF phenotypes (Fig 9A and 9B). Interestingly, a patch clamp analysis of the effects of the MTSBn modification at site F78, showed opposite results relative to those observed *in vivo;* treatment with MTSBn produced a statistically significant increase in the pressure threshold needed to gate the channels (treated/untreated 1.19 ± 0.01, n = 5, p<0.005 paired Student t-test).

### The C-terminal domain

The effect of cysteine substitutions on MscL’s channel activity *in vivo* was studied for the second transmembrane domain TM2, (residues K101-A114). Of the 13 residues included in this region 2 showed a lower viability due to a GOF phenotype (S1F Fig and [23]).

**Fig 10:**
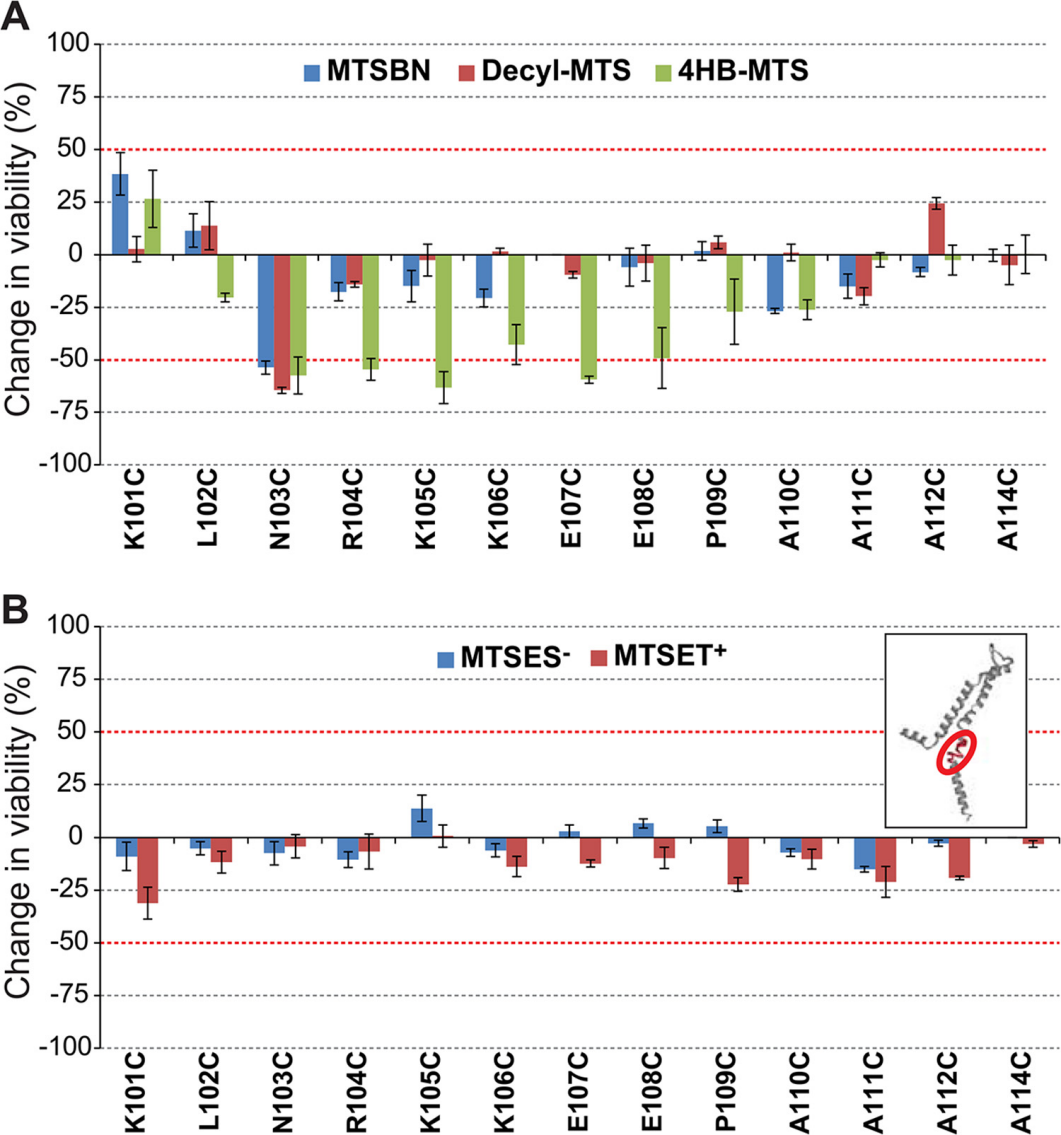
Effects of post translational modifications on the C-terminal domain of MscL determined by *in vivo* channel activity. The viability of the osmotically fragile strain MJF455 expressing MscL cysteine substituted mutants from residues K101 to A114 (insert), was measured after an osmotic down-shock. The graphs show the differences in viability between non-treated and post-transnationally modified channels with (A) the hydrophobic MTS reagents MTSBn (blue), decyl-MTS (red) or 4HB-MTS (green) or (B) the negatively charged MTSES^-^ (blue) and positively charged MTSET^+^ (red). The red grid line indicates a ± 50% change that was used as a threshold for further studies.

Post-translational modification in this region with the tyrosine like MTS reagent 4HB-MTS changed the viability of N103C to E108C more than 50% when compared to osmotic down-shock only. N103C viability was also affected by the presence of MTSBn and decyl-MTS (Fig 10A). No changes were observed in this region when shocked in the presence of the charged probes MTSES^-^ or MTSET^+^ (Fig 10B).

**Fig 11:**
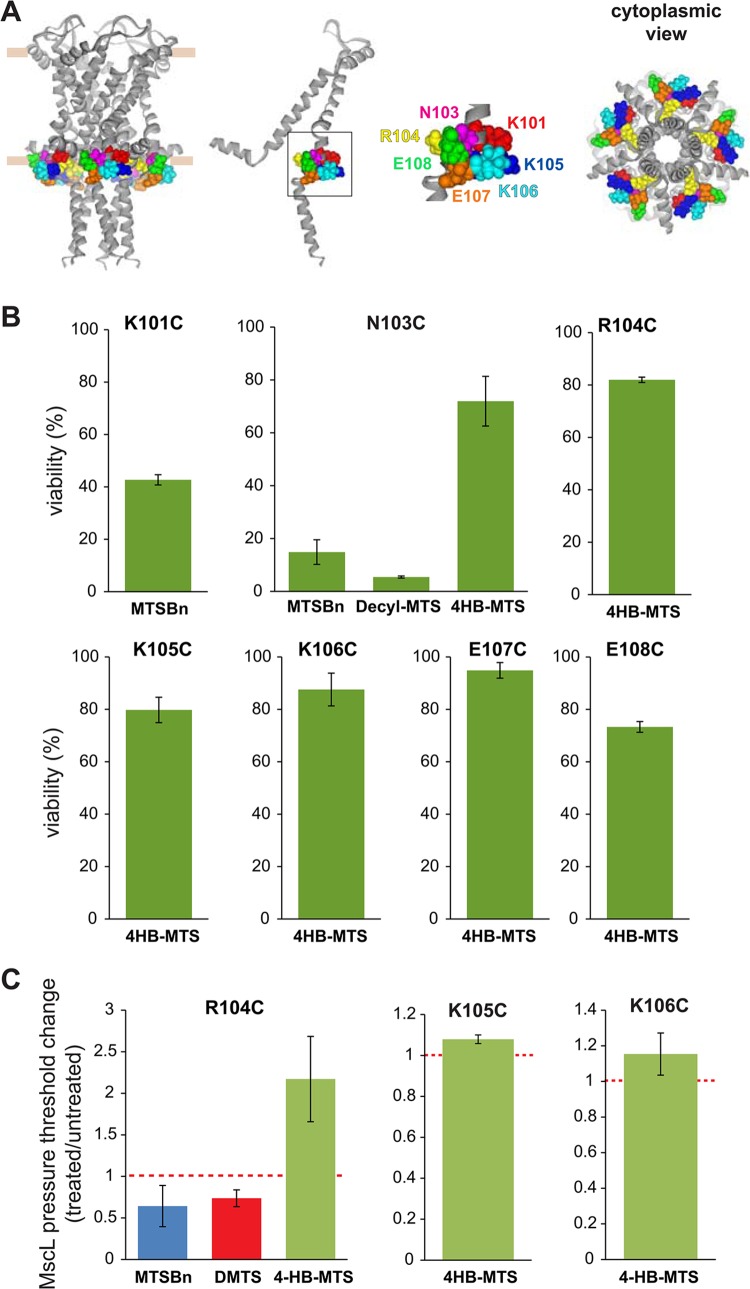
Functional changes by substitutions in the C-terminal domain of MscL, determined by *in vivo* and patch clamp experiments. (A) The location of the residues showing changes in viability upon post-translational modifications is highlighted in the closed structure of *E*. *coli* MscL. From left to right: a pentameric MscL is shown in a side view where the approximate location of the membrane marked, a single subunit and a close up of the region with the labeled residues followed by a cytoplasmic view. (B) Viability MJF 367 (*mscL* ^-^) cells shock in the presence of the indicated MTS reagent is shown for each individual MscL mutant. C. The changes in the pressure threshold required to gate R104C, K105C and K106C MscL caused by MTS reagents is graphed as the ratio between before and after modification of the same patch. The red line indicates no change.

To determine which channel phenotype caused the observed changes in the viability, secondary screens were performed. MTSBn substitution at residue K101 led to a decrease in viability while 4HB-MTS substitutions from R104-E108 did not (Fig 11A and 11B). As previously reported, N103 showed a dichotic behavior with MTSBn and decyl-MTS effecting a GOF phenotype, but not 4HB-MTS apparently because of its movement into the membrane upon channel gating [14].

The patch clamp results shown in Fig 11C correlate with the *in vivo* results, with 4HB-MTS increasing the pressure threshold needed to gate R104C, K105C and K106C. Interestingly MTSBn and decyl-MTS have opposite effect on residue R104, a pattern similar to the one observed for its neighbor residue N103 [14].

## Discussion

The molecular mechanisms underlying the gating of mechanosensitive channels is a field of increasing interest since these channels are involved in essential physiological processes such as vasoregulation, touch, hearing, pain, depression, and responses to ischemia. Interestingly, mechanosensitive channels from eukaryotic and prokaryotic organisms are found in structurally unrelated protein families. For example, the few eukaryotic mechanosensitive channels known to date belong to very different families like the two pore potassium channels (TRAAK, TREK-1, and TREK-2), the transient receptor potential family (TRPV4) and the Piezo channels [24–26]. The prokaryotic mechanosensitive channels MscL and MscS are also structurally unrelated. However, some of these channels have common mechanisms of activation including modulation by amphipaths such as LPC and arachidonic acid, and there are also some analogous and common structural domains in otherwise very different families. For example an α- helix running along the cytoplasmic membrane and connected to the pore domain plays a crucial role in the gating of such different channels as TRPY1, TRAAK, TREK, MscL and MscS [2, 8, 27–30]. This highlights the relevance of a detailed understanding of the gating mechanisms of mechanosensitive channels.

The choice of the *in vivo* screens as the main approach to determine MscL channel activity has the double advantage of giving relevant information about channel function in its native environment and comparing channel activity before and after post-translational modifications. These post-translational modifications at the site of the cysteine substituted residue confer it with different properties and their effects on channel activity can be directly evaluated. Furthermore, the possibility to combine bacterial screens using different bacterial strains allows one to distinguish between different channel phenotypes and better understand the transitions of MscL domains during gating. It should be pointed out that negative results are difficult to interpret; a lack of effect could mean that the site is tolerant of changes, or it could simply be that the residue is buried and inaccessible to the reagents. This could, for instance, explain why there are only 2 hits within TM2, which is predicted to have ample protein-lipid and protein-protein interactions, and both hits were toward the cusp of the domain. However, the predictions of large conformational changes occurring upon gating combined with a pore size large enough to easily pass the reagents to the cytoplasmic side would suggest that accessibility should be better than typical membrane proteins. Indeed, the assay identified several residues predicted to be inaccessible. One of the more extreme examples is L96, toward the cytoplasmic end of TM2, which was influenced by the charged MTSET^+^ and MTSES^-^ reagents; presumably this could only occur if the reagents crossed the open pore to gain cytoplasmic accessibility.

Early in the study of MscL, the N-terminal domain was shown to play a crucial role in channel gating since deletion of just 11 amino acids lead to a non-functional channel [10]. From the first analysis of the crystal structure of the *M*. *tuberculosis* MscL, this region was not resolved, but later in a reanalysis of the crystallographic data it was solved as a α- helix running along the cytoplasmic membrane [3, 31]. Molecular dynamic simulation using this newly defined structure embedded in lipids, first proposed that the S1 region remains parallel to the membrane during MscL gating [32]. A systematic study of the disulfide bridging pattern, *in vivo* and patch clamp data agreed with the proposed location of the N-terminal domain. From these data, we proposed a model in which this region would act as a sensor and stabilizer of the TM1 domain during channel opening [8]. Further studies using a combination of patch clamp, fluorescence resonance energy transfer (FRET) spectroscopy, electron paramagnetic resonance experiments, and molecular and Brownian dynamics simulations, suggest a similar role for this region [16]. Interestingly, an amphipathic α-helix connected to the pore domain by a flexible glycine, is found in various mechanosensitive channels as diverse as TRAAK, TREK-1, TREK-2 [29, 30, 33], the yeast transient receptor potential channel, TRPY1 [27], and the bacterial mechanosensitive channel MscS [2, 3].

Although several cysteine substitutions in the N-terminal region lead to altered channel phenotypes (S1 Fig A), very few of the post-translational modifications produced changes in viability (Fig 2A and 2B). This lack of observed effect may be due to the fact that the N-terminal domain is densely packed with the cytoplasmic region of TM2 at the lipid interface [34]. This conformation could either interfere with the accessibility of MTS probes to the sites, or make the region more sensitive to the size of the probes. One such example is residue E9, which leads to a GOF phenotype when substituted to cysteine. Interestingly E9 is in close proximity to residue K101 as determined by disulfide trapping experiments, and favoring this interaction leads to a locked closed channel [34]. Although it has also been shown that electrostatic interactions between E9 and K101 are crucial for channel gating [23], we failed to see a remediation of E9C GOF phenotype when treated with MTSES^-^. As stated earlier, this lack of an effect could be due to inaccessibility, or to the bulkier MTS substitution affecting channel function in this tightly packed region. Hydrophobic substitutions at A11 and M12 conferred upon the channels a GOF phenotype (Fig 3B). As confirmed by direct study of the channels activity by patch clamp experiments, hydrophobic post-translational modifications in M12C with decyl and MTSBn, significantly lowered the channels pressure threshold (Fig 3D). This decrease in pressure threshold after a hydrophobic substitution suggests that while the channel opens these residues move to a more hydrophobic environment, which could be the lipid membrane since these residues are located at the cytoplasmic membrane interphase of TM1.

The R13 to D18 region, sometimes called the S1-TM1 linker of MscL, is highly conserved and has been proposed to contain a very conserved motive (NhhD), part of a common ancestral sensor for both voltage and membrane tension [35, 36]. Interestingly, residues N15 and D18 in Eco MscL have been proposed essential for the formation of a water chain across the channel that leads to and stabilizes the opening of MscL [32]. Cysteine substitutions of the most conserved residues in this domain, including R13, G14 and D18, produced very strong phenotypes when mutated to cysteines, causing a reduction in viability of about 90% (S1A Fig). R13C is a strong GOF mutant reducing growth by 70% and G14C and D18C are strong LOF mutants [7]. Surprisingly, modification of the cysteines with the MTS reagents used in this study did not significantly modify these phenotypes, as determined by our *in vivo* screen. In one model, the R13 residues are proposed to approach their counterparts in neighboring subunits during gating with the electrostatic repulsion acting as a resistive force to gating (Levin and Blount, 2004); the finding that neither MTSET^+^ nor MTSES^-^ sustitutions appear to remediate the GOF phenotype in our screens, suggests that either the residue is inaccessible, or that the model may be an over simplification. In another detailed model for the gating of *E*. *coli* MscL which proposed the tilting of the transmembrane domains during opening, G14 was suggested to act as a hinge in the transmission of tension between S1 and TM1[37, 38], so it may not be surprising that few if any substitutions at this location are tolerated. Interestingly, G14 is the hinge between the cytoplasmic amphipathic α-helix and the pore region, part of the common structural feature shared by many otherwise structurally unrelated mechanosensitive channels.

The pore forming first transmembrane domain of MscL was found to be a hot mutational spot in a random mutagenesis study where many mutations, almost all to more hydrophilic residues, led to more sensitive or GOF channels [22]. A subsequent study mutated a single residue, G22, to all other amino acids and confirmed that at least for this site, G22, the GOF phenotype correlated with the hydrophilicity of the residue [39]. There are several lines of data that suggest this domain twists in a clockwise (as seen from the periplasm) corkscrew fashion upon channel opening [5, 19, 40, 41]. Furthermore, it has been proposed that a “hydrophobic lock” at the constriction point of the pore stabilizes the closed state of MscL and it is the transient exposure of these residues to an aqueous environment upon channel gating that is the primary energy barrier [13]. Cysteine substitutions in the cytoplasmic part of TM1 effected only GOF phenotypes (S1B Fig), [7]; for residue V23C, the proposed constriction point, post-translational modification with hydrophobic probes reverted this phenotype (Fig 4A). A similar effect has been described for residue G26C [19, 20, 42], for which a longer time of treatment with decyl-MTS leads to a non-functional channel, probably due to an unbreakable hydrophobic lock when more subunits are substituted. As shown for the V23C MscL (S2 Fig), the behavior in patch of this mutated channel is complicated, presumably due to disulfide bridging in the *in vitro* redox environment. This is true for other TM1 mutants including R13C and G26C, presumably due to their close proximity in the pentameric structure. It is interesting to point out that, in the case V23C, treatment with MTS reagents to cross-linked channels is indeed effective as seen for the reversion of the channel phenotypes, implying that the probes are able to break these cysteine bonds (S2 Fig).

The periplasmic loop of MscL is the least conserved part of the channel and probably the least studied. Early work showed that enzymatic digestion of the periplasmic loop, periplasmic and cytoplasmic extra membrane domains of MscL, or reconstitution of separately synthesized TM1 and TM2 domains, all lead to more sensitive channels that retained their mechanosensitive. These studies suggested that these domains tuned the mechanosensitivity level of the channel [43, 44]. Consistent with this work, it was also found that select mutations at Q65 could lead to either GOF or LOF phenotypes [45]. In addition, a molecular dynamic simulation predicts that the loop region is highly mobile, and attributes this region a role in the stability of the open state as well as structural stability during the tilting of the membranes during gating [32]. Cysteine substitution in this region caused few but strong altered phenotypes. One interesting residue in the periplasmic loop region, Q56, is also known to modulate the kinetics of MscL, with aromatic substitutions drastically increasing the channel mean open time [10]. Our results agree with this previous observation, with the aromatic substitutions decreasing the viability of the cells (Fig 6A), although the change did not reach the 50% cut of established for this work. Interestingly, recent work using chimeras between MscL orthologues, found that residue I49 plays a critical role in channel kinetics, acting as a “clasp knife spring” controlling the stability of the open and closed channel states of the channel [46]. At the neighboring residue L48, post-translational modification with decyl-MTS, effected a strong LOF phenotype, probably because the anchoring of the protein at the membrane at this site impairs the mobility required to gate MscL.

The second transmembrane domain of MscL surrounds TM1, and is in close contact with the lipids. One of the hits in this region was I96C treated with either MTSET^+^ or MTSES^-^. This residue has been previously shown to yield a GOF phenotype when mutated to a positive residue, and is predicted to interact with V23 upon channel gating [41]. But perhaps the most interesting residue within this domain is F78, which is predicted to face the membrane, and has been proposed as a membrane tension sensor in an asparagine scan of the periplasmic interface of TM2 [47]. Among the most conserved residues in this region are residues G76, F78, F85 and F93 [48], but interestingly cysteine substitutions have little (F78, F85) or the opposite effect on channel function (G76C), than the asparagine substitutions. While the F78C mutated protein had little if any phenotype *in vivo*, treatment with MTSBn, which should reconstitute the aromatic ring to the site, led to a GOF phenotype. The fact that the disulfide linkage adds bulk to the residue and is not a strict reconstitution of the original structure can also account for this effect. To further complicate the interpretation, the channel phenotype, as assayed by patch clamp, appeared to be a LOF when treated with MTSBn. These data might be explained by subtle differences between the *in vivo* assay and patch clamp including the membrane potential and a potential asymmetry between the two leaflets of the lipid bilayers *in vivo* that may be lost upon spheroplasts preparation, which could contribute to the discrepancies. Given the differences between the *in vivo* and patch clamp experiments it is surprising that in most instances the data from these two experimental approaches correlate well.

The cytoplasmic region of MscL at the lipid-cytoplasmic interphase has been proposed to enter a more hydrophobic environment to accommodate for the tilting of TM2 while channel gating [14, 23]. We have proposed that a conserved charge cluster acts as a “knot in a rope” to stop the tilting and incorporation of the helix in the membrane Although deletion studies have shown that charges in the conserved RKKEE region (residues 104–108) are essential for channel function, our data suggest that no single residue plays a critical function and instead it appears to be the property of the entire cluster that is important (S1E Fig). In our previous study, we concentrated on N103 and found a correlation between hydrophobicity of the substitution and channel sensitivity. Furthermore, fluorescence quenching measurements in the presence of brominated lipids suggested that the residue approaches the lipid membrane during channel gating. Here we further find that the residue adjacent to N103, R104, displays a similar behavior in patch clamp experiments, with the very hydrophobic substitutions by MTSBn and decyl-MTS leading to more sensitive channels and the tyrosine like substitution to less sensitive channels (Fig 11C). Interestingly, restoration of, by post-translational modification with the charged MTS probes, did not have an effect in channel activity *in vivo* (Fig 10B). Again the tight packing of this region might either impair probe accessibility or be too sensitive to the size of the probe.

Although MscL is probably the best studied MS channel, the lack of an open crystal structure makes predicting the changes that occur upon gating difficult. However, because of the conformational changes are large, leading to what is the largest gated pore known, many residues and sub-domains drastically change their local environment upon gating. Hence, studies in which the fundamental properties of specific residues are changed can give valuable clues for transition and open states. In the present work, using *in vivo* screens we identify important functional domains and residues. These assays allow a very interesting perspective since they compare the protein’s different domains under identical experimental conditions, thus giving a functional map of the protein and a better understanding of which residues are critical for channel function, as well as highlighting MscL domains that undergo the more drastic environmental changes upon gating.

## Supporting Information

S1 Fig Effect of single cysteine substitutions on MscL channel function *in vivo*. The viability after an osmotic down-shock of MJF 455 cells expressing single cysteine mutants is shown for substitutions in (A) the N-terminal region, (B) the first transmembrane domain (TM1), (C) the periplasmic loop, (D) the second transmembrane domain (TM2) and (E) the C-terminal domain of *E*. *coli* MscL. The red dotted line is for reference that indicates the viability after an osmotic down-shock of MJF 455 cells expressing wild type MscL. Red asterisks highlight mutants whose decrease in viability is due to a gain of function phenotype. Each bar represents the mean of at least three independent experiments. Error bars represent standard error.
(EPS)

S2 Fig Effect of post translational modifications on V23C MscL channel activity in patch clamp. A. The changes in the pressure threshold required to gate V23C MscL after MTS treatment, are presented as the ratio between the pressure threshold before and after modification of the same patch. The red line indicates no change. B. Representative traces of V23C MscL and before (control) and after treatment with MTSBn and decyl-MTS. The negative pressure applied to the patch is under each trace on the right. *p<0.007 paired Student t-test n≥7.
(EPS)
